# The relevance of the intrinsic subtype to the clinicopathological features and biomarkers in Japanese breast cancer patients

**DOI:** 10.1186/1477-7819-11-293

**Published:** 2013-11-18

**Authors:** Masako Tamaki, Takako Kamio, Shingo Kameoka, Noriko Kojimahara, Toshio Nishikawa

**Affiliations:** 1 Department of Surgery II, Tokyo Women’s Medical University, 8-1 Kawada-cho, Shinjuku-ku, Tokyo 162-8666, Japan; 2 Department of Hygiene and Public Health II, Tokyo Women’s Medical University, 8-1 Kawada-cho, Shinjuku-ku, Tokyo 162-8666, Japan; 3 Department of Pathology, Tokyo Women’s Medical University, 8-1 Kawada-cho, Shinjuku-ku, Tokyo 162-8666, Japan

## Abstract

**Background:**

Breast cancer is a disease rich in diversity, and it can be categorized into the immunohistochemical intrinsic subtypes : ER/PR + and HER2-, ER/PR + and HER2+, HER2 type, basal-like and unclassified.

**Methods:**

In this study, in addition to the clinicopathological features potentially associated with the intrinsic subtypes, protein expression and genetic mutations of key molecules associated with breast cancer prognosis and treatment sensitivity were analyzed. The distribution of subtypes in the patient population and the differences in marker distribution across the subtypes were investigated.

**Results:**

The immunohistochemical features of 471 consecutive surgical cases of women with primary breast cancer, treated in a single institution, were examined. There were 306 patients who were ER/PR + HER2- (65%); 41 who were ER/PR + HER2+ (8.7%); 59 with HER2 type (12.5%); 37 with basal-like (7.9%); and 28 patients whose breast cancer was unclassified (5.9%). There were no significant differences between the subtypes regarding age, menopausal status, disease stage, lymphatic invasion, blood vessel invasion and lymph node metastasis. Statistically significant differences were found for histological type and grade. Regarding protein expression and genetic mutation, significant differences were found in the distribution within each subtype for six out of 12 molecules investigated.

**Conclusions:**

This study revealed that subtypes differ not only in their clinical pathological profiles, such as histological types and histological grades, but also in molecular expression. The molecular expression patterns observed for each intrinsic subtype may help the selection of an optimal treatment strategy.

## Background

Breast cancer is a disease rich in diversity, and it can be categorized into subtypes with distinct biological features. Sorlie *et al*. showed that breast cancer can be divided into five ‘intrinsic subtypes’ by gene expression analysis using a DNA microarray [1]. Subsequently, Carey *et al*. reported that breast cancers can be divided into the five intrinsic subtypes by a protein expression analysis of estrogen receptor (ER), progesterone receptor (PR), human epidermal growth factor receptor type 2 (HER2), epidermal growth factor receptor (EGFR) and cytokeratin 5 or 6 (CK5/6), using immunohistochemistry (IHC) [2]. This IHC-intrinsic subtyping is now widely used [3-6].

The biological characteristics and the clinical presentations differ between the five intrinsic subtypes [1-6]. Intrinsic subtypes can be useful as reference data in the existing treatment selection process. Moreover, the development of new treatments for breast cancer may need to take into consideration the intrinsic subtypes. Distribution data, or prevalence (the proportion of each subtype in the patient population), and knowledge of the clinicopathological features associated with each subtype are being accumulated regarding intrinsic subtypes [1-6]. However, there have so far been few reports on biomarker protein expression and genetic mutation profiles for the intrinsic subtypes, particularly in Japanese populations. Therefore, in this study, the distribution of key molecules associated with breast cancer prognosis and treatment sensitivity was investigated, in addition to subtype distribution and associated clinicopathological characteristics. More specifically, the protein expression of phosphatase and tensin homolog deleted on chromosome 10 (PTEN), insulin-like growth factor-1 receptor (IGF-1R), B-cell lymphoma 2 (Bcl-2), c-Kit, hepatocyte growth factor receptor (c-Met), hypoxia-inducible factor 1-alpha (HIF-1α), alpha-type platelet-derived growth factor receptor (PDGFRA), survivin, vascular endothelial growth factor receptor 2 (VEGFR2), vascular endothelial growth factor A (VEGF-A), in addition to ER, PR, HER2, EGFR, CK5/6, and *PIK3CA* genetic mutation were analyzed. And the differences in biomarker distribution among the subtypes were investigated. PTEN loss and expression of c-Kit, c-Met, HIF-1α, PDGFRA VEGFR2, and VEGF-A have been reported to be worse prognostic factors for breast cancer, whereas expression of IGF-1R, Bcl-2 and survivin are reportedly good prognostic factors for breast cancer.

Ethnic differences have been reported in the prevalence of intrinsic subtypes and associated clinicopathological features [2], and so it is desirable to accumulate information on the targeted ethnic group when selecting or developing treatments. In Japan, there have so far been only a few reports that have investigated the prevalence of intrinsic subtypes. The results of this study are expected to make a significant contribution to the growing body of data regarding Japanese patients.

## Methods

### Patients

Candidates for this investigation comprised 471 consecutive surgical cases of women with primary breast cancer who were treated in the Department of Surgery II, Tokyo Women’s Medical University throughout July 2004 to November 2007, excluding patients in whom pre-surgical treatment was performed. In cases of bilateral breast cancer, each tumor was separately set as a sample, and for cases of multiple breast cancer, only the main tumor was set as a sample and investigated.

### Ethical approval

This study protocol was approved by the Ethical Committee of Tokyo Women's Medical University. This is an observational study by using stored samples and existing medical records. Only investigators in Tokyo Women’s Medical University have been allowed to access data with personal information.

### Methods

Clinicopathological characteristics (menopausal status, disease stage, histological type, histological grade, tumor size, lymphatic invasion, blood vessel invasion, lymph node metastasis), protein expression and genetic mutation were investigated. The differences in distribution between each subtype were statistically examined and analyzed. Key biomarkers in breast cancer prognosis and treatment sensitivity were selected, and their protein expression was measured by IHC. Gene mutation in *PIK3CA* exons 9 and 20 was detected by PCR amplification followed by direct sequencing.

### Protein expression analysis

In addition to ER, PR, HER2, EGFR and CK5/6, which are required for subtyping, 10 other molecules reported to be predictive for breast cancer prognosis and sensitivity to treatment were assessed: PTEN, IGF-1R, Bcl-2, c-Kit, c-Met, HIF-1α, PDGFRA, survivin, VEGFR2 and VEGR-A. For HER2, fluorescence *in situ* hybridization was done when IHC was scored as 2+. CK5/6 IHC was performed on only the triple negative breast tumors (ER-,PR- and HER2-). The assay conditions and scoring criteria of IHC assays are shown in the Table 1.

**Table 1:** Experimental conditions of immunohistochemistry assays

**Antibody** (**clone**)	**Dilution**	**Source**	**Criteria for positive**/**negative**
ER : Antibody (ID5)	×100	Dako	Positive: Stained cells >10%
PR : Antibody (PgR636)	×500	Dako	Positive: Stained cells >10%
HER2 (HercepTest)	Ready to use	Dako	HercepTest criteria
EGFR (EGFR pharmDx kit)	Ready to use	Dako	EGFR pharmDx kit criteria
CK5/6 (D5/16 B4)	×100	Dako	Positive: Stained cells >10%
PTEN (6H2.1)	1:100	Dako	- No staining
			+ Decreased staining intensity
			++ Equal or increased staining intensity
IGF-1R (C-20)	1:50	Santa Cruz	Score 0, 1+, 2+, 3+ using HercepTest criteria
Bcl-2 (124)	×50	Dako	Positive: Stained cells >10%
c-Kit (polyclonal; CD117)	×500	Dako	Positive: Stained cells >10%
c-Met (C-28, polyclonal)	1:100	IBL	Positive: Stained cells >10%
HIF-1α (H1alpha67)	×100	Novus Biologicals	Positive: Stained cells >10%
PDGFRA (C20, polyclonal)	1:200	Thermo	Positive: Stained cells >10%
Survivin (12C4)	×200	Novus Biologicals	Positive: Stained cells >20%
VEGFR2 (polyclonal; Flk-1(A-3))	×100	Santa Cruz	Positive: Stained cells >10%
VEGF-A (VG1)	Ready to use	Thermo	Positive: Stained cells >10%

ER: estrogen receptor, PR: progesterone receptor, HER2: human epidermal growth factor receptor type 2, EGFR: epidermal growth factor receptor, CK5/6: cytokeratin 5 or 6, PTEN: phosphatase and tensin homolog deleted on chromosome 10, IGF-1R: insulin-like growth factor-1 receptor, Bcl-2: B-cell lymphoma 2, c-Met: hepatocyte growth factor receptor, HIF-1α: hypoxia-inducible factor 1-alpha, PDGFRA: alpha-type platelet-derived growth factor receptor, VEGFR2: vascular endothelial growth factor receptor 2, VEGF-A: vascular endothelial growth factor A.

NOTE. Dako: Tokyo, Japan. Santa Cruz: Tokyo, Japan. IBL: Gunma, Japan. Novus Biologicals: Littleton, Colorado, USA. Thermo: Waltham, Massachusetts, USA.

Intrinsic subtypes were assigned as follows: ER/PR + HER2- (ER + and/or PR+, and HER2-); ER/PR + HER2+ (ER + and/or PR+, and HER2+); HER2 type (ER-, PR- and HER2+); basal-like (triple negative; ER-, PR-, HER2-, and EGFR+ and/or CK5/6+); and unclassified (ER-, PR-, HER2-, EGFR- and CK5/6).

### Mutation analysis

Genomic DNA was extracted from paraffin-embedded tumor tissue sections using DEXPAT® (Takara Bio Inc., Shiga Japan). Genomic DNA was amplified by PCR using the specific amplifying primers for exons 9 and 20 of *PIK3CA*. The mRNA sequence for *PIK3CA* was obtained from GenBank [GenBank:NM_006218]. Since exon 20 is relatively large (271 bp), the first and second halves of the gene were separately amplified with one overlapping region of 57 base pairs. The purified PCR products were sequenced using BigDye® Terminator Cycle Sequencing Kit (Applied Biosystems, Foster City, CA, USA) and analyzed with an automated capillary ABI PRISM 3100 genetic analyzer (Applied Biosystems). The DNA chromatograms were analyzed using the SeqScape® ver2.5 software (Applied Biosystems) for any single mutations, followed by visual confirmation. All mutations identified were confirmed by a second analysis by independent PCR amplification and direct sequencing. Genomic DNA from corresponding normal tissue was subjected to sequence analysis to confirm that the nucleotide substitutions detected in tumor tissues were somatic rather than germline mutations.

### Statistical methods

Analysis of variance, Fisher’s exact probability test and Cochran-Mantel-Haenszel test were performed to evaluate the statistical significance of any variation in biomarker prevalence and clinicopathological features within the subtypes, using SAS software version 9.1.3. For the statistical differences between each group, a *P*-*value* under 0.05 was regarded as statistically significant. Exact odds ratio and 95%CI adjusted by age strata (except for menopausal status) were estimated by using SAS 9.1.3 Freq procedure.

## Results

The clinicopathological features for each intrinsic subtype as determined by the expression of ER, PR, HER2, EGFR and CK5/6 are shown in Table 2. Out of the 471 patients, there were 306 who were ER/PR + HER2- (65.0%), 41 who were ER/PR + HER2+ (8.7%), 59 of HER2 type (12.5%), 37 of basal-like (7.9%) and 28 patients of unclassified (5.9%).

**Table 2:** Clinicopathological features for intrinsic subtypes

	**All cases**	**ER/PR+, HER2-**	**ER**/**PR+, HER2+**	**HER2 type**	**Basal-like**	**Unclassified**	***P***
**Cases (%)**	471 (100)	306 (65.0)	41 (8.7)	59 (12.5)	37 (7.9)	28 (5.9)	
**Mean age at diagnosis (years)**	57.3 ±12.6	57.1 ±12.9	55.2 ±8.7	58.5 ±12.0	57.6 ±13.7	59.9 ±13.8	0.57 (Analysis of variance)
**Menopausal status at diagnosis**							0.8 (Fisher)
Premenopausal	169 (35.9)	115 (37.6)	15 (36.6)	18 (30.5)	11 (29.7)	10 (35.7)	
Postmenopausal	302 (64.1)	191 (62.4)	26 (63.4)	41 (69.5)	26 (70.3)	18 (64.3)	
**Stage**							0.1 (Cochran-Mantel-Haenszel)
0	57 (12.1)	41 (13.4)	2 (4.9)	8 (13.6)	1 (2.7)	5 (17.9)	
I	138 (29.3)	97 (31.7)	16 (39.0)	9 (15.2)	9 (24.3)	7 (25.0)	
IIA, IIB	232 (49.3)	139 (45.4)	19 (46.3)	34 (57.6)	24 (64.9)	16 (57.1)	
IIIA, IIIB, IIIC	40 (8.5)	27 (8.8)	4 (9.8)	7 (11.9)	2 (5.4)	0 (0)	
IV	4 (0.8)	2 (0.7)	0 (0)	1 (1.7)	1 (2.7)	0 (0)	
**Histological type**							<0.01 (Fisher)
Papillotubular carcinoma	94 (20.0)	49 (16.0)	13 (31.7)	18 (30.5)	7 (18.9)	7 (28.0)	
Solid-tubular carcinoma	83 (17.6)	36 (11.8)	6 (14.6)	14 (23.7)	23 (62.2)	4 (14.3)	
Scirrhous carcinoma	194 (41.2)	145 (47.4)	19 (46.4)	18 (30.5)	3 (8.1)	9 (32.1)	
Noninvasive carcinoma, Paget’s disease	65 (13.8)	49 (16.0)	2 (4.9)	8 (13.6)	1 (2.7)	5 (17.9)	
Others	35 (7.4)	27 (8.8)	1 (2.4)	1 (1.7)	3 (8.1)	3 (10.7)	
**Histological type (invasive ductal carcinoma only)**							<0.01 (Fisher)
Papillotubular carcinoma	94 (25.3)	49 (21.3)	13 (34.2)	18 (36.0)	7 (21.2)	7 (35.0)	
Solid-tubular carcinoma	83 (22.4)	36 (15.7)	6 (15.8)	14 (28.0)	23 (69.7)	4 (20.0)	
Scirrhous carcinoma	194 (52.3)	145 (63.0)	19 (50.0)	18 (36.0)	3 (9.1)	9 (45.0)	
**Lymphatic invasion**							0.31 (Fisher)
Positive	269 (57.1)	169 (55.2)	27 (65.9)	31 (52.5)	26 (70.3)	16 (57.1)	
Negative	202 (42.9)	137 (44.8)	14 (34.1)	28 (47.5)	11 (29.7)	12 (42.9)	
**Blood vessel invasion**							0.19 (Fisher)
Positive	33 (7.0)	18 (5.9)	4 (9.8)	8 (13.6)	1 (2.7)	2 (7.1)	
Negative	438 (93.0)	288 (94.1)	37 (90.2)	51 (86.4)	36 (97.3)	26 (92.9)	
**Lymph node metastases (Level I)**							0.62 (Fisher)
Positive	88 (18.7)	59 (19.3)	7 (17.1)	14 (23.7)	5 (13.5)	3 (10.7)	
Negative	383 (81.3)	247 (80.7)	34 (82.9)	45 (76.3)	32 (86.5)	25 (89.3)	
**Lymph node metastases (Level II)**							0.97 (Fisher)
Positive	30 (6.4)	19 (6.2)	3 (7.3)	4 (6.8)	2 (5.4)	2 (7.1)	
Negative	441 (93.6)	287 (93.8)	38 (92.7)	55 (93.2)	35 (94.6)	26 (92.9)	
**Lymph node metastases (Level III)**							0.56 (Fisher)
Positive	16 (3.4)	11 (3.6)	3 (7.3)	1 (1.7)	1 (2.7)	0 (0)	
Negative	455 (96.6)	295 (96.4)	38 (92.7)	58 (98.3)	36 (97.3)	28 (100)	
	**Invasive carcinoma cases**	**ER/PR+, HER2-**	**ER**/**PR+, HER2+**	**HER2type**	**Basal-like**	**Unclassified**	***P***
**Cases (%)**	406 (100%)	257 (63.3%)	39 (9.6%)	51 (12.6%)	36 (8.9%)	23 (5.7%)	
**Tumor size**							0.54 (Fisher)
≤ 2.0 cm	272 (67.0)	179 (69.6)	24 (61.5)	32 (62.7)	24 (66.7)	13 (56.5)	
>2.0 cm	134 (33.0)	78 (30.4)	15 (38.5)	19 (37.3)	12 (33.3)	10 (43.5)	
**Histological grade (total score)**							<0.01 (Fisher)
Grade I	200 (49.3)	148 (57.6)	21 (53.8)	14 (27.4)	5 (13.9)	12 (52.2)	
Grade II or III	201 (49.5)	106 (41.2)	18 (46.2)	36 (70.6)	31 (86.1)	10 (43.5)	
Unknown	5 (1.2)	3 (1.2)	0 (0)	1 (2.0)	0 (0)	1 (4.3)	

There was no significant difference across the subtypes in age, menopausal status, disease stage, lymphatic invasion, blood vessel invasion or lymph node metastasis (Level I to III). Significant differences in the distribution between the intrinsic subtypes was observed when the cancer was divided into five histological types, namely papillotubular carcinoma, solid-tubular carcinoma, scirrhous carcinoma, noninvasive carcinoma/Paget's disease, and others. Scirrhous carcinoma was more frequent in ER/PR + HER2- and ER/PR + HER2+ cases, at 47% and 46%, respectively. Papillotubular carcinoma, scirrhous carcinoma and solid-tubular carcinoma occurred at almost the same frequency for HER2 type, and the frequency of solid-tubular carcinoma was higher for patients with basal-like IHC compared to other groups, at 62%. Moreover, there was a significant difference among the intrinsic subtypes in the distribution of three histological types, namely papillotubular carcinoma, solid-tubular carcinoma and scirrhous carcinoma (*P* <0.01). When tumor size and histological grades of the 406 cases of invasive carcinoma, excluding noninvasive carcinoma and Paget’s disease, were compared among intrinsic subtypes, no significant differences were observed in tumor size. Significant differences (*P* <0.01) were observed between the subtypes for the histological grade (Grade I compared to Grades II and III combined).

**Figure 1:**
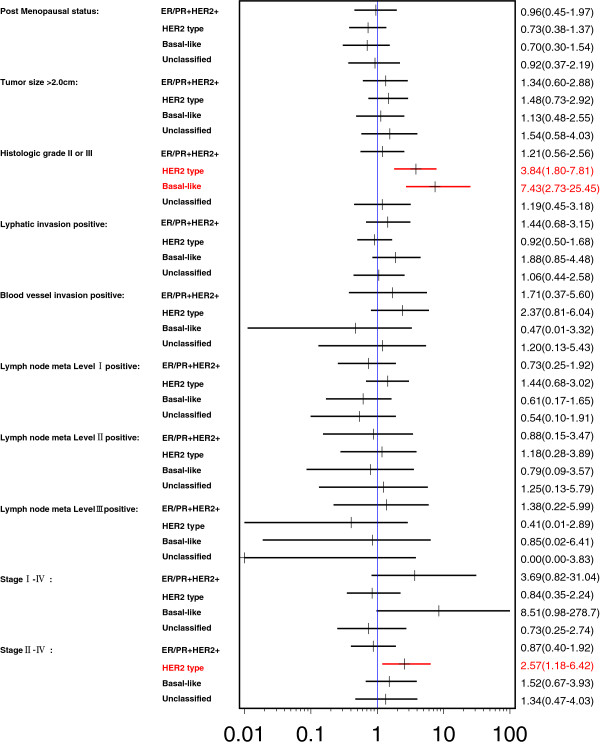
**Odds ratios (95% confidence interval) of clinicopathological features for intrinsic subtypes (reference: ER/PR + HER2-).**

Higher histologic grade (Grade II/III) was strongly associated with HER2 type (odds ratio (OR) 3.84; 95% CI 1.80, 7.81) and basal-like type (OR 7.43; 95% CI 2.73, 25.45) compared to the reference ER/PR+ Her2- type (Figure 1). More specifically, the histological grade of ER/PR + HER2-, in contrast with HER2 type and basal-like, was strongly associated with lower histologic grade (Grade I).

**Figure 2:**
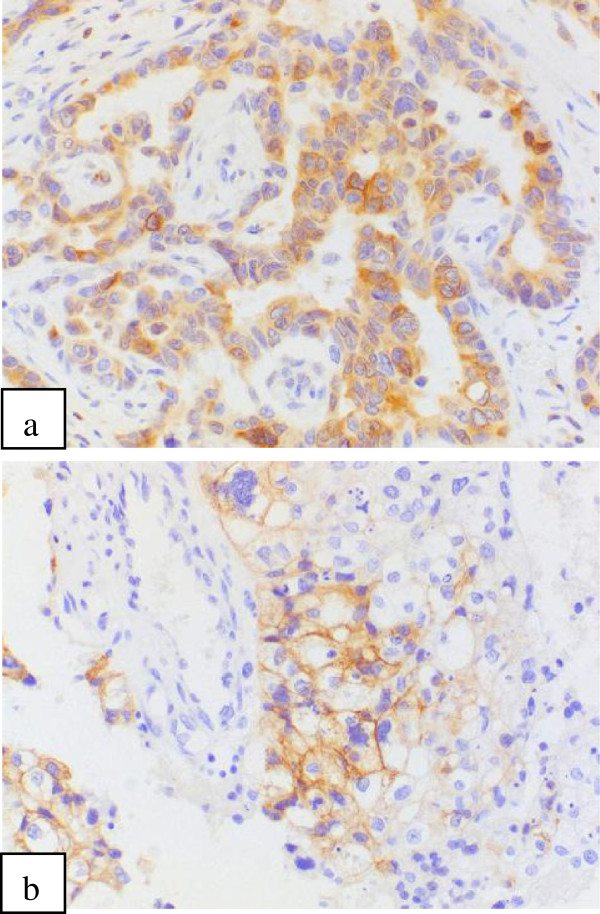
**Immunohistochemistry. a** Bcl-2(B-cell lymphoma 2) positive x200. **b** c-Kit positive × 200.

Protein expression and genetic mutation overall and by each intrinsic subtype are shown in Table 3. Overall, EGFR was expressed by 16.0% of patients, PTEN expression was reduced in 50.9% of patients and IGF-1R expression was 85.9%. Bcl-2 expression (Figure 2a) was 65.0% and c-Kit expression (Figure 2b) was 7.1%; however, there was no c-Kit expression observed in patients who were ER/PR + HER2+. Overall, c-Met expression was 72.6%, and HIF-1α expression was 34.1%. The expression of PDGFRA was 86.9%. The expression of survivin was 82.4%, VEGFR2 was 68.1% and VEGF-A was 60.4%. Significant differences were observed between each subtype for EGFR positivity, PTEN reduction, Bcl-2 positivity, c-Kit, c-Met and survivin. Exon 9 of *PIK3CA* was difficult to measure with paraffin blocks, so there were only 91 cases of successful measurements. Out of those, somatic mutation was observed in eight cases (8.8%). In exon 20 of *PIK3CA*, somatic mutation was observed in 28 (7.8%) out of 357 measurable cases.

**Table 3:** Protein expression and genetic mutation by intrinsic subtypes

	**All cases**	**ER/PR + HER2-**	**ER/PR + HER2+**	**HER2 type**	**Basal-like**	**Unclassified**	***P***
**Cases (%)**	471 (100)	306 (65.0)	41 (8.7)	59 (12.5)	37 (7.9)	28 (5.9)	
**EGFR**							<0.01
Positive	74 (16.0)	16 (5.4)	5 (12.5)	19 (32.8)	34 (91.9)	0 (0)	
Negative	388 (84.0)	283 (94.6)	35 (87.5)	39 (67.2)	3 (8.1)	28(100)	
Missing	9	7	1	1			
**PTEN**							<0.01
Reduced	234 (50.9)	156 (52.7)	18 (43.9)	16 (27.6)	28 (75.7)	16 (57.1)	
Normal	226 (49.1)	140 (47.3)	23 (56.1)	42 (72.4)	9 (24.3)	12 (42.9)	
Missing	11	10		1			
**IGF-1R**							0.77
Positive	397 (85.9)	255 (85.6)	36 (87.8)	50 (86.2)	30 (81.1)	26 (92.9)	
Negative	65 (14.1)	43 (14.4)	5 (12.2)	8 (13.8)	7 (18.9)	2 (7.1)	
Missing	9	8		1			
**Bcl-2**							<0.01
Positive	303 (65.0)	249 (83.0)	27 (67.5)	7 (12.1)	6 (16.2)	14 (50.0)	
Negative	160 (35.0)	51 (17.0)	13 (32.5)	51 (87.9)	31 (83.8)	14 (50.0)	
Missing	8	6	1	1			
**c-Kit**							<0.01
Positive	33 (7.1)	15 (5.0)	0 (0)	7 (12.1)	10 (27.0)	1 (3.6)	
Negative	433 (92.9)	287 (95.0)	41 (100)	51 (87.9)	27 (73.0)	27 (96.4)	
Missing	5	4		1			
**c-Met**							0.03
Positive	328 (72.6)	203 (69.5)	34 (82.9)	47 (85.5)	28 (75.7)	16 (59.3)	
Negative	124 (27.4)	89 (30.5)	7 (17.1)	8 (14.5)	9 (24.3)	11 (40.7)	
Missing	19	15		4		1	
**HIF-1α**							0.76
Positive	159 (34.1)	110 (36.4)	12 (29.3)	18 (31.0)	11 (29.7)	8 (28.6)	
Negative	307 (65.9)	192 (63.6)	29 (70.7)	40 (69.0)	26 (70.2)	20 (71.4)	
Missing	5	4					
**PDGFRA**							0.49
Positive	393 (86.9)	255 (87.6)	34 (85.0)	52 (91.2)	30 (81.1)	22 (81.5)	
Negative	59 (13.1)	36 (12.4)	6 (15.0)	5 (8.8)	7 (18.9)	5 (18.5)	
Missing	19	15	1	2		1	
**Survivin**							0.02
Positive	383 (82.4)	240 (79.5)	34 (85.0)	56 (96.6)	30 (81.1)	23 (82.1)	
Negative	82 (17.6)	62 (20.5)	6 (15.0)	2 (3.4)	7 (18.9)	5 (18.9)	
Missing	6	4	1	1			
**VEGFR2**							0.59
Positive	318 (68.1)	206 (68.0)	25 (61.0)	44 (75.9)	25 (67.6)	18 (64.3)	
Negative	149 (31.9)	97 (32.0)	16 (39.0)	14 (24.1)	12 (32.4)	10 (35.7)	
Missing	4	3		1			
**VEGF-A**							0.14
Positive	279 (60.4)	180 (60.0)	25 (61.0)	42 (72.4)	18 (48.6)	14 (50.0)	
Negative	183 (39.6)	118 (40.0)	16 (39.0)	16 (27.6)	19 (51.4)	14 (50.0)	
Missing	9	8		1			
***PIK3CA *** **exon 9**							0.94
Mutated	8 (8.8)	6 (9.7)	0 (0)	1 (10.0)	0 (0)	1 (14.3)	
Wild type	83 (91.2)	56 (90.3)	6 (100)	9 (90.0)	6 (100)	6 (85.7)	
Missing	380	45	35	49	31	22	
***PIK3CA *** **exon 20**							0.17
Mutated	28 (7.8)	20 (8.7)	4 (14.3)	1 (2.2)	3 (10.3)	0 (0)	
Wild type	329 (92.2)	211 (91.3)	24 (85.7)	44 (97.8)	26 (89.7)	24 (100)	
Missing	114	75	13	14	8	4	

EGFR: epidermal growth factor receptor, PTEN: phosphatase and tensin homolog deleted on chromosome 10, IGF-1R: insulin-like growth factor-1 receptor, Bcl-2: B-cell lymphoma 2, c-Met: hepatocyte growth factor receptor, HIF-1α: hypoxia-inducible factor 1-alpha, PDGFRA: alpha-type platelet-derived growth factor receptor, VEGFR2: vascular endothelial growth factor receptor 2, VEGF-A: vascular endothelial growth factor A.

**Figure 3:**
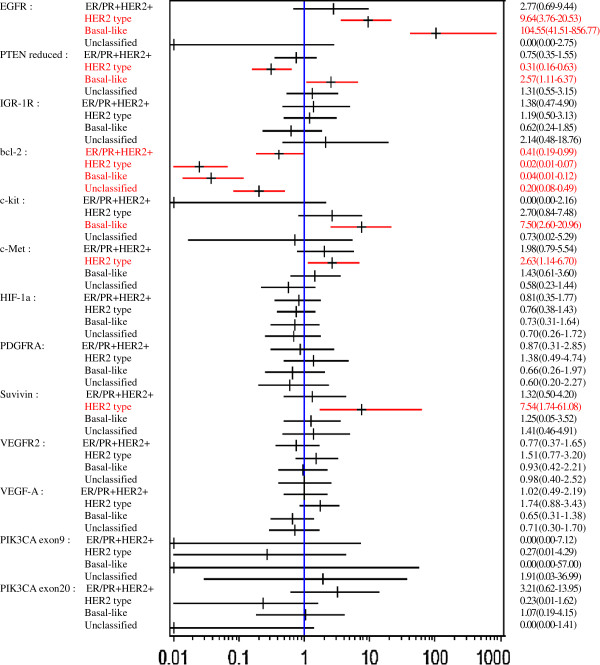
**Odds ratios (95% confidence interval) of biomarker expressions according to intrinsic subtypes (reference: ER/PR + HER2-).**

Figure 3 shows associations between biomarker expressions and intrinsic subtypes with odds ratios when ER/PR + HER2- is set as a reference group. EGFR expression was strongly associated with HER2 type (OR 9.64; 95% CI 3.76, 20.53) and basal-like type (OR 104.55; 95% CI 41.51, 856.77). PTEN reduction was associated with basal-like type (OR 2.57; 95% CI 1.11, 6.37) and inversely associated with HER2 type (OR 0.31; 95% CI 0.16, 0.63). Bcl-2 expression was inversely associated with all the subtypes including ER/PR + HER2+, HER2 type, basal-like and unclassified types. c-Kit expression was associated with basal-like type, and c-Met and survivin expressions were associated with HER2 type.

When each biomarker expression was compared between each subtype, with ER/PR + HER2- as a reference (Table 4), the expression of Bcl-2 was lower in ER/PR + HER2+, and expression of c-Kit was not observed. The PTEN reduction and Bcl-2 expression were lower in HER2 type, and the expression of EGFR, c-Met and survivin was significantly higher. The expression of EGFR and c-Kit and the PTEN reduction were significantly higher in the basal-like type and expression of Bcl-2 was significantly lower. The expression of Bcl-2 was significantly lower in the unclassified type.

**Table 4:** Comparison of molecular profile of each intrinsic subtype

	**ER/PR + HER2-**		**ER/PR + HER2+**		**HER2 type**		**Basal-like**		**Unclassified**	
	**n**	**OR**	**n**	**OR**	**n**	**OR**	**n**	**OR**	**n**	**OR**
**EGFR**										
Positive	16	1.0 (Ref)	5	2.77 (0.69, 9.44)	19	9.64 (3.76, 20.53)	34	104.55 (41.51, 856.77)	0	0.00 (0.00, 2.75)
Negative	283		35		39		3		28	
**PTEN**										
Reduced	156	1.0 (Ref)	18	0.75 (0.35, 1.55)	16	0.31(0.16, 0.63)	28	2.57 (1.11, 6.37)	16	1.31 (0.55, 3.15)
Normal	140		23		42		9		12	
**Bcl-2**										
Positive	249	1.0 (Ref)	27	0.41 (0.19, 0.99)	7	0.02 (0.01, 0.07)	6	0.04 (0.01, 0.12)	14	0.20 (0.08, 0.49)
Negative	51		13		51		31		14	
**c-Kit**										
Positive	15	1.0 (Ref)	0	0.00 (0.00, 2.16)	7	2.70 (0.84, 7.48)	10	7.50 (2.60, 20.96)	1	0.73 (0.02, 5.29)
Negative	287		41		51		27		27	
**c-Met**										
Positive	203	1.0 (Ref)	34	1.98 (0.79, 5.54)	47	2.63 (1.14, 6.70)	28	1.43 (0.61, 3.60)	16	0.58 (0.23, 1.44)
Negative	89		7		8		9		11	
**Survivin**										
Positive	240	1.0 (Ref)	34	1.32 (0.50, 4.20)	56	7.54 (1.74, 61.08)	30	1.25 (0.05, 3.52)	23	1.41 (0.46, 4.91)
Negative	62		6		2		7		5	

Results are odds ratio (OR) adjusted by age and 95% confidence interval.

EGFR: epidermal growth factor receptor, PTEN: phosphatase and tensin homolog deleted on chromosome 10, Bcl-2: B-cell lymphoma 2, c-Met: hepatocyte growth factor receptor.

The expression of biomarkers whose expression significantly differed between subtypes, was compared with that of ER, PR, HER2 and EGFR, on which subtyping was made (Table 5). PTEN reduction showed a significant difference between HER2 positive and negative groups, and was significantly higher in the reduced expression group (*P* < 0.01). Expression of Bcl-2 was significantly higher in ER positive group (*P* < 0.01), PR positive group (*P* < 0.01), HER2 negative group (*P* < 0.01), and EGFR negative group (*P* < 0.01), respectively, compared to the referent groups. c-kit expression was significantly lower in ER positive group (*P* < 0.01), PR positive group (p < 0.01), and EGFR negative group (*P* < 0.01), respectively, compared to the referent groups. c-Met expression was positive rate significantly higher in HER2 positive group (*P* < 0.01) and EGFR positive group (*P* =0.046). The survivin positive rate showed significant differences between ER positive and negative, and HER2 positive and negative groups, and was significantly higher in ER negative group (*P* =0.03), and HER2 positive group (*P* < 0.01).

**Table 5:** **Expression of ER**, **PR**, **HER 2 and EGFR by biomarkers whose expression significantly differed between subtypes**

	**ER**		***P***	**PR**		***P***	**HER2**		***P***	**EGFR**		***P***
**Biomarkers**	**Positive** **(%)**	**Negative** **(%)**		**Positive (%)**	**Negative (%)**		**Positive** **(%)**	**Negative** **(%)**		**Positive** **(%)**	**Negative** **(%)**	
**PTEN**			0.76			0.10			<0.01			0.80
Reduced	163 (50.3)	71 (52.2)		134 (47.7)	100 (55.9)		34 (34.3)	200 (55.4)		39 (52.7)	195 (50.9)	
Normal	161 (49.7)	65 (47.8)		147 (52.3)	79 (44.1)		65 (65.7)	161 (44.6)		35 (47.3)	188 (49.1)	
**Bcl-2**			<0.01			<0.01			<0.01			<0.01
Positive	268 (82.0)	35 (25.7)		238 (83.2)	65 (36.7)		34 (34.7)	269 (73.7)		20 (27.4)	279 (72.5)	
Negative	59 (18.0)	101 (74.3)		48 (16.8)	112 (63.3)		64 (65.3)	96 (26.3)		53 (73.4)	106 (27.5)	
**c-Kit**			<0.01			<0.01			1.00			<0.01
Positive	15 (4.5)	18 (13.2)		11 (3.8)	22 (12.3)		7 (7.1)	26 (7.1)		16 (21.6)	16 (4.1)	
Negative	315 (95.5)	118 (86.8)		276 (96.2)	157 (87.7)		92 (92.9)	341 (92.9)		58 (78.4)	371 (95.9)	
**c-Met**			0.42			0.16			<0.01			0.046
Positive	229 (71.3)	99 (75.6)		194 (70.0)	134 (76.6)		81 (84.4)	247 (69.4)		60 (82.2)	267 (70.8)	
Negative	92 (28.7)	32 (24.4)		83 (30.0)	41 (23.4)		15 (15.6)	109 (30.6)		13 (17.8)	110 (29.2)	
**Survivin**			0.03			0.06			<0.01			0.24
Positive	263 (80.0)	120 (88.2)		228 (79.7)	155 (86.6)		90 (91.8)	293 (79.8)		64 (87.7)	314 (81.1)	
Negative	66 (20.0)	16 (11.8)		58 (20.3)	24 (13.4)		8 (8.2)	74 (20.2)		9 (12.3)	73 (18.9)	

Fisher's exact probability test was used for statistical analysis.

ER: estrogen receptor, PR: progesterone receptor, HER2: human epidermal growth factor receptor type 2, EGFR: epidermal growth factor receptor, PTEN: phosphatase and tensin homolog deleted on chromosome 10, Bcl-2: B-cell lymphoma 2, c-Met: hepatocyte growth factor receptor.

## Discussion

This study describes the distribution of the IHC-intrinsic tumor subtypes, ER/PR + HER2-, ER/PR + HER2+, HER2 type, basal-like and unclassified, among Japanese patients with breast cancer. Clinicopathological characteristics of each intrinsic subtype are also presented. The present study further provides new evidence of the biomarker expression profile of each intrinsic subtype, which has scarcely been examined elsewhere.

### Distribution of intrinsic subtypes

Distribution of intrinsic subtypes in this study with the proportion of ER/PR + HER2- (65.0%), ER/PR + HER2+ (8.7%), HER2 type (12.5%), basal-like (7.9%) and unclassified (5.9%) was not essentially different from those reported elsewhere, as shown in Table 6 that shows subtype distributions in different populations [2,3,6].

**Table 6:** Comparison of intrinsic subtypes distribution in different populations

**Reference**	**Country**	**All patients (%)**	**ER/PR + HER2-**	**ER/PR + HER2+**	**HER2 type**	**Basel-like**	**Unclassified**
**Our research**	Japan	471 (100)	306 (65.0)	41 (8.7)	59 (12.5)	37 (7.9)	28 (5.9)
**Shibuta *et al*. [**[6]**]**	Japan	4,266 (100)	3,046 (71)	321 (8)	398 (9)	501 (12)	
**Carey *et al*. [**[2]**]**	US	496 (100)	255 (51.4)	77 (15.5)	33 (6.7)	100 (20.1)	31 (6.3)
	African American	196 (100)	93 (47.4)	25 (12.8)	16 (8.2)	52 (26.5)	10 (5.1)
	Non-African American	300 (100)	162 (54)	52 (17.3)	17 (5.7)	48 (16)	21 (7)
**Tamimi *et al*. [**[3]**]**	US	2,249 (100)	1,650 (73)	116 (5)	128 (6)	245 (11)	110 (5)

### ER/PR + HER2- subtype

In our investigation, ER/PR + HER2- had a lower histological grade, higher Bcl-2 expression and lower EGFR expression compared to the other subtypes. This profile may play a role in the better prognosis reported for the ER/PR + HER2-.

The histological grade of ER/PR + HER2- was the lowest among the five subtypes, and it was significantly lower than for the HER2 and basal-like types. Among biomarkers measured, Bcl-2 expression was significantly higher in ER/PR + HER2- than the other subtypes, whereas EGFR expression was as low as 5.4%, which was significantly lower than HER2 and basal-like types. EGFR expression was reported to be an independently worse prognostic factor for breast cancer [7], whereas Bcl-2 expression is known to be a favorable prognostic factor for breast cancer.

Bcl-2 expression has been reported to correlate with ER expression [7], and to be inversely correlated with EGFR expression, HER2 expression and histological grade [7,8]. The better prognosis that is seen for Bcl-2-positive breast cancer had thus been attributed to its higher prevalence of ER expression; however, Dawson *et al*. [9] demonstrated Bcl-2 expression to be associated with a good prognosis independent of ER expression, by comparing the associations between Bcl-2 expression and the prognosis of an ER positive group with that of an ER negative group separately.

### ER/PR + HER2+ subtype

There was no significant difference in the clinicopathological features between ER/PR + HER2- and ER/PR + HER2+ in this investigation. The prevalence of Bcl-2 expression in ER/PR + HER2+ was lower than that in ER/PR + HER2-. Dawson *et al*. [9] demonstrated both Bcl-2 negativity and HER2 positivity to be associated with a worse prognosis, and the group with the profile of Bcl-2 negative combined with HER2 positive showed the shortest survival. Taken together, the prognosis of ER/PR + HER2+ is thus expected to be worse than that of ER/PR + HER2-, and this has, in fact, been reported [5,6].

### HER2 type

When the clinicopathological characteristics of HER2 type were examined, higher histological grade (Grade II/III compared to Grade I) was found to be significantly associated with HER2 type compared to ER/PR + HER2-. Kurebayashi *et al*. [5] and Shibuta *et al*. [6] also reported a significant difference between the subtypes of histological grades and the nuclear grades.

In the current study, HER2-overexpressing tumors had a higher histologic grade, a higher positive rate of c-Met, survivin and EGFR, and a lower prevalence of PTEN reduction and Bcl-2 than ER/PR + HER2-. These differences in the molecular characteristics between ER/PR + HER2- and HER2 type may contribute to the reported worse prognosis of HER2 type in comparison to ER/PR + HER2- [2,4-6].

The HER2 signaling pathway is related to EGFR, PTEN, IGF-1R, c-Met and *PIK3CA*. Among these biomarkers, our data demonstrated the proportion of PTEN reduction to be significantly lower whereas the expression of EGFR and c-Met was significantly higher in HER2 type than in ER/PR + HER2-. However, no significant differences between subtypes were observed in expression of IGF-1R or *PIK3CA* mutation frequency. Moreover, in this investigation, Bcl-2 expression was significantly lower and the survivin expression was significantly higher in HER2 type.

The Phosphoinositide 3-kinase-Akt also known as protein kinase B or PKB pathway is downstream of HER2 and is activated by *PIK3CA* mutation and PTEN loss [10,11]. *PIK3CA* mutation is reported to be observed in 25% to 40% of all breast cancers. In this investigation, *PIK3CA* mutation in exon 20 in the HER2 type was found in only one out of the 45 measurable cases (2.2%). PTEN reduction was observed in 27.6% of the HER2 type cases, and the proportion was smaller than that for ER/PR + HER2-. Capodanno *et al*. [10] and Perez-Tenorio *et al*. [12] also reported the proportion of PTEN reduction to be smaller in the group with HER2 overexpression compared to luminal types.

c-Met is known to be involved in several biological functions, including cell proliferation, cell survival, angiogenesis, cancer cell invasion and metastasis. c-Met has been reported to be frequently expressed in HER2 positive breast cancers, and in this investigation also, c-Met positivity was high in HER2 type (85.5%) and ER/PR + HER2+ type (82.9%). Regarding sensitivity to treatment, there have been reports suggesting that c-Met expression may be involved in resistance to trastuzumab [13], and thus the c-Met expression status should be considered when selecting the optimal treatment for HER2 type cases.

The EGFR positive rate of HER2 type was the second highest after basal-like type among the subtypes. EGFR positivity is reported to be a worse prognostic factor, and is also reported to be high in HER2 positive breast cancer [14-21].

The prevalence of Bcl-2 expression in HER2 type was significantly lower than that in ER/PR + HER2-. Dawson *et al*. [9] demonstrated Bcl-2 positivity to be a favorable prognostic factor independent of HER2 positivity.

The positive rate of survivin was 96.6%. There are many reports describing frequent expression of survivin in cancer cells, with a prevalence ranging from 60% to 80% in breast cancers [13,22]. Survivin is an apoptosis-suppressing factor, and is reported to be an independent factor for a poor prognosis [22]. In our study, survivin positive rate was relatively high in the HER2 positive group, ER negative group and PR negative group (Table 5). Ryan *et al*. also reported the survivin positive rate to be as high as 90% and its expression level was higher in ER negative, PR negative and HER2 positive breast cancers [23]. Asanuma *et al*. [16] also found the survivin positive rate to be higher in HER2 positive cancer and furthermore showed survivin expression to be more frequent in EGFR positive cancer.

In summary, compared to ER/PR + HER2-, HER2 type showed a higher histologic grade, higher positive rates of c-Met, survivin and EGFR, and a lower prevalence of PTEN reduction and Bcl-2. These differences in the molecular characteristics between ER/PR + HER2- and HER2 type may explain the reported worse prognosis of HER2 type in comparison to ER/PR + HER2- [2,4-6].

### Basal-like type

In our investigation, histological grade of basal-like type was significantly higher than for ER/PR + HER2-, which was concordant with past reports [2-6]. The distribution of each histologic type among basal-like type breast tumors showed the frequency of solid-tubular carcinoma to be the highest at 62.2%, whereas scirrhous carcinoma was most prevalent among ER/PR + HER2- and ER/PR+ HER2+. Nakajima *et al*. [24] also reported the most prevalent histologic type in ER/PR + HER2- and ER/PR + HER2+ to be scirrhous carcinoma, while the frequency of solid-tubular carcinoma was as high as that of scirrhous carcinoma at 34% in basal-like type [24]. A higher proportion of solid-tubular carcinoma in tumors of a basal-like subtype is also consistent with results by Tsuda *et al*., who found EGFR positivity in solid-tubular carcinoma to be 17% - much higher than that in scirrhous carcinoma (6%) and papilla-tubular carcinoma (3%) [14]. It must be noted that EGFR expression was one of the criteria for the basal-like subtype classification in this study, and 91.9% of basal-like cases were positive for EGFR.

The prevalence of PTEN reduction in basal-like type tumors was significantly higher than that for ER/PR + HER2- in this study. López-Knowles *et al*. [25] also observed the rate of PTEN reduction to be higher than for the other subtypes, and Marty *et al*. [26] reported that PTEN reduction was more prevalent in basal-like type compared to HER2 type.

Regarding Bcl-2 expression in basal-like type tumors, the positive rate was significantly lower than that for ER/PR + HER2- in this study. c-Kit expression has been reported to be observed in almost all normal breast tissues, while it is frequently diminished or lost in breast tumor tissues [27]. The c-Kit positive rate in breast cancer was reported as 14% by Nielson *et al*. [28] and 14.7% by Charpin *et al*. [24]. In our study, c-Kit expression was observed in 7.1% of patients overall. Its positive rate in basal-like type tumors was 27.0%, which was significantly higher than that in ER/PR + HER2-. Our results showing the c-Kit positive rate to be higher in ER negative, PR negative and EGFR positive groups (Table 5) are consistent with the difference in the c-Kit positive rate between basal-like and ER/PR + HER2-. Tsuda *et al*. [14] reported a similar relationship of c-Kit expression with ER, PR and EGFR expressions. c-Kit expression is also known to be a worse prognostic factor for breast cancer [29].

From the above, there is a possibility that the characteristics of basal-like type, specifically its higher histological grades, higher rate of PTEN reduction, lower rate of Bcl-2 positivity and higher rate of c-Kit positivity compared to ER/PR + HER2- type, may together help to explain why the prognosis of basal-like type is poorer than that for ER/PR + HER2- [2,4-6]. Moreover, since PTEN reduction is reported to correlate with Akt activity [26], the inhibition of Akt activation or suppression of c-Kit signals could thus become a target for treatment for basal-like type breast cancer.

### Unclassified type

In this study, out of the triple negative (ER-, PR- and HER2-) cases, EGFR- and CK5/6- cases were considered to have an unclassified subtype. As reported above, the histological grade of basal-like tumors was significantly higher than those of ER/PR + HER2-, however there was no difference between unclassified and ER/PR + HER2-. A higher nuclear grade of basal-like triple-negative tumors compared with non-basal-like triple negative (the unclassified type in our study) was observed by Yamamoto *et al*. [30] as well.

The expression of Bcl-2 in the unclassified subtype was significantly lower compared to ER/PR + HER2-; however, it was higher than that of basal-like type.

Positive IGF-1R expression was higher in the unclassified group than in ER/PR + HER2- (OR 2.14; 95% CI 0.48, 18.76) though it was not significant, whereas that of basal-like type was lower than that for ER/PR + HER2-. Hartog *et al*. reported the expression of IGF-1R to be a worse prognostic factor in triple-negative breast tumors [31].

A lower expression of Bcl-2 and a higher expression of IGF-1R in unclassified tumors compared to ER/PR + HER2- were thus suggested to be partly involved in the reported worse prognosis of unclassified type cancer than ER/PR + HER2- [2,4,5].

Although IGF-1R expression was a worse prognostic factor in triple-negative breast tumors, it was a favorable prognostic factor in ER positive breast tumors [31]. IGF-1R expression thus could be used for accurately selecting patients who are best indicated to undergo IGF-1R-targeted therapy.

## Conclusion

In this study, we clarified not only that the clinicopathological profiles, such as histological types and histological grades, vary between the breast cancer subtypes, but also that there are differences in the molecular expression profiles between subtypes. The reported differences in the prognosis between intrinsic subtypes may therefore be partly attributable to the differences in their molecular characteristics. Furthermore, the molecular characteristics of each subtype can be used as reference data when developing new treatments for breast cancer and for selecting patients who are sensitive or resistant to each therapy.

## Abbreviations

bp: Base pair; Bcl-2: B-cell lymphoma 2; CK5/6: Cytokeratin5/6; c-MET: Hepatocyte growth factor receptor; EGFR: Eepidermal growth factor receptor; ER: Estrogen receptor; HER 2: Human epidermal growth factor receptor type 2; HIF-1α: Hypoxia-inducible factor 1-alpha; IGF-1R: Insulin-like growth factor-1 receptor; IHC: Immunohistochemistry; OR: Odds ratio; PCR: Polymerase chain reaction; PDGFRA: Alpha-type platelet-derived growth factor receptor; PR: Progesterone receptor; PTEN: Phosphatase and tensin homolog deleted on chromosome 10; VEGFR2: Vascular endothelial growth factor receptor 2; VEGR-A: Vascular endothelial growth factor A; PIK3CA: Phosphatidylinositol-3-kinase, catalytic, alpha.

## Competing interests

This study was conducted as a sponsored study of GlaxoSmithKline K.K. with its full financial support.

## Authors’ contributions

MT,TK,SK were involved in the review of literature, acquisition of data and drafting and completing the manuscript. NK participated in the design of the study and statistical analysis. TN carried out histopathological analysis. All authors read and approved the final manuscript.
